# Triboelectric Generator Based on Oriented Self-Assembled Peptide Microbelts

**DOI:** 10.3390/nano12223955

**Published:** 2022-11-10

**Authors:** Vladislav Slabov, João Vidal, Pavel Zelenovskii, Svitlana Kopyl, Marco P. Soares dos Santos, Andrei Kholkin

**Affiliations:** 1 Department of Physics & CICECO—Aveiro Institute of Materials, University of Aveiro, 3810-193 Aveiro, Portugal; 2 Department of Mechanical Engineering & TEMA, University of Aveiro, 3810-193 Aveiro, Portugal; 3 Department of Chemistry & CICECO—Aveiro Institute of Materials, University of Aveiro, 3810-193 Aveiro, Portugal

**Keywords:** diphenylalanine, TENG, self-assembly

## Abstract

Along with piezoelectric nanogenerators, triboelectric nanogenerators (TENGs) collecting energy from mechanical vibrations proved to be simple, low-cost, and efficient sources of electricity for various applications. In view of possible biomedical applications, the search for TENGs made of biomolecular and biocompatible materials is demanding. Diphenylalanine (FF) microstructures are promising for these applications due to their unique characteristics and ability to form various morphologies (microribbons, spherical vesicles, fibrils, micro- and nanotubes, nanorods, etc.). In this work, we developed a contact-separate mode TENG based on arrays of oriented FF microbelts deposited by dip-coating technique and studied their performance under various temperature treatments. We show that these TENGs outperform piezoelectric nanogenerators based on FF microbelts in terms of short-circuit current (I_SC_), open-circuit voltage (V_OC_), and output power. It was found that bound water captured in FF nanochannels mainly affects V_OC_, whereas mobile water increases I_SC_. We also found that the cyclization of FF molecules increases the performance of TENG likely due to an increase in surface energy and surface flattening.

## 1. Introduction

The rapid development of autonomous wireless sensor networks for various medical, environmental, and engineering applications leads to constantly increasing demand for long-life and low-power energy sources [1,2,3]. Energy harvesting recently arose as a cutting-edge technology for low-cost renewable “green” energy collection from vibrations, friction, temperature, pressure variations, optical irradiation, etc. [4,5,6,7,8,9,10,11,12,13]. Among the numerous harvesting systems suggested so far, the triboelectric nanogenerators (TENGs) collecting energy from the periodic movements proved themselves to be simple, low-cost, and effective for various multiscale applications [13]. Regardless, the comprehensive theory of the triboelectric effect is still far from completeness [14], the TENGs are already used nowadays for self-powering in a wide range of technologies [15,16]. The triboelectric pair is a key element of any TENG. The choice of the pair should be based on the source of the harvested mechanical energy. It is interesting to note that humans provide a large amount of mechanical energy during their daily lives, which could be converted by TENGs into a significant amount of electric power [17,18,19]. Therefore, permanent contact between the TENG and the body requires the use of biocompatible materials. Recently, several natural and eco-friendly materials such as leaves, paper, proteins, etc., demonstrated the ability to harvest electric energy from mechanical power sources ensuring low-cost production [20]. One of the self-assembling peptides, diphenylalanine (FF), has attracted significant research interest due to the wide variety of microstructures provided by FF self-organization and the appearance of piezoelectric response that is comparable with traditional inorganic piezoelectrics [21,22,23,24,25]. Different deposition techniques such as dip-coating, inkjet printing, and physical vapor deposition [26,27,28] allow controlling of the geometry of the FF microstructures including microribbons, spherical vesicles, fibrils, micro- and nanotubes, rods, etc. [27,29,30]. Some of these microstructures were already investigated for piezoelectric and triboelectric harvesting [26,31,32]. FF microtubes could provide additional functionality to the TENG due to two types of water molecules confined in the nanochannels [33]. The chemisorbed bound water directly interacting with the peptide backbone provides its structural integrity, whereas mobile water influences the functional response of FF nanotubes [33,34]. Thus, the control of the degree of filling of the nanochannels allows the tuning of piezoelectric and pyroelectric properties of the nanotubes. In this work, we developed the contact-separate mode TENG based on FF microbelts prepared by the dip-coating technique and studied its harvesting performance under various temperature treatments. We demonstrated the effect of the water inside the nanochannels on its triboelectric properties.

## 2. Materials and Methods

### 2.1. Microbelts Fabrication

The microbelts were grown using a dip-coating method. The lyophilized powder of diphenylalanine (H-L-Phe-L-Phe-OH, FF) was purchased in Bachem (Bubendorf, Switzerland). 1,1,1,3,3,3-hexafluoro-2-propanol (HFIP) was used to prepare the stock solution with an FF concentration of 2 mg/mL. The stock solution was then mixed with deionized water at a ratio of 3:1 to achieve the final concentration of FF 0.5 mg/mL. These concentrations were chosen to prevent the self-assembly of microtubes in the bulk solution during the coating procedure. Such a mixture could be stored for several days without noticeable precipitation or transparency changes. However, a fresh mixture was prepared before each experiment to avoid aggregation and better control the concentration. The 10×30 mm^2^ ITO/PET substrates were dipped into the mixture and pulled up at various velocities, namely 30, 40, and 50 µm/min by ND-DC 11/1 75 Dip Coater.

### 2.2. Characterization Techniques

The surface morphology of the grown FF microstructures was investigated by Nikon Eclipse LV150 optical microscope, NTMDT Solver NEXT atomic force microscope (AFM), and Hitachi TM 4000 scanning electron microscope (SEM). The ImageJ software was used to determine the area covered by microstructures. 

The grown microstructures were characterized by powder X-ray diffraction (XRD) and Fourier-transform infrared (FTIR) spectroscopy. Powder XRD patterns were collected at room temperature (around 298 K) in standard Bragg–Brentano configuration using a Panalytical Empyrean diffractometer equipped with a Cu Kα1 radiation source (λ = 0.15406 nm), a linear PIXEL detector with fixed divergence slit of 1/2°, and a spinner sample holder. The measurements were performed in the range 3–40° (2θ) using a continuous counting method with 0.026° step size and 200 s of counting time. FTIR spectra were measured using a Bruker Optics Tensor 27 spectrometer (Billerica, MA, USA) coupled to a horizontal attenuated total reflectance (ATR) cell, using 256 scans at a resolution of 4 cm^−1^ in the range between 450 and 4000 cm^−1^.

Differential scanning calorimetry (DSC) measurements were performed using a NETZSCH thermal analyzer coupled with a quadrupole mass spectrometer NETZSCH QMS 403 providing detection of molecules in the mass range from 1 to 300 m z^−1^.

### 2.3. Triboelectric Harvesting Measurements

Macroscopic triboelectric energy harvesting measurements were carried out in a contact-separation mode using a conventional shaker-voltmeter setup [35]. The Teflon part of the triboelectric generator was fixed to a solid surface, and the FF/PET part was attached to the support of the shaker TV 50018 (TIRA GmbH, Schalkau, Germany). A 10 Hz square-wave voltage signal from a functional generator FG410 (Yokogawa, Musashino shi, Japan) amplified by a power amplifier BAA 60 (TIRA GmbH), was used to drive the shaker with a displacement amplitude of about 1 mm, acceleration amplitude of about 0.2 g, and frequency of 10 Hz. The excitation acceleration was measured using a piezoelectric shear accelerometer M352C66 (PCB Piezotronics Inc., Depew, NY, USA), mounted on the shaker’s support, a signal conditioner 480C02 (PCB Piezotronics Inc.), and a lock-in amplifier HF2LI (Zurich Instruments AG, Zurich, Switzerland). The harvested voltage between the strained triboelectric plates characterized by complex amplitude, δV, was measured using a pre-amplifier stage before the lock-in amplification. This pre-amplifier provides a voltage divider followed by a very high input impedance (>100 TΩ) operational amplifier (ADA4530-1) operating as a voltage buffer. All measurements were controlled by a customized user interface based on LabVIEW.

## 3. Results

### 3.1. Sample Fabrication and Surface Characterization

**Figure 1:**
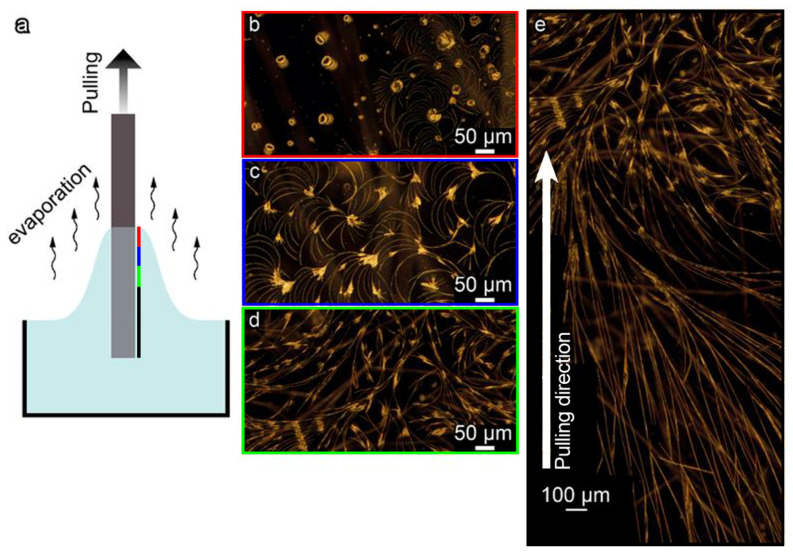
FF microstructures fabrication by the dip-coating method: (**a**) schematic of the process; (**b**–**d**) optical dark-field images of the microstructures formed at the beginning of the process; (**e**) microstructures transformation from disordered crystals to quasi-regular elongated belts. Color frames indicate the position at the substrate where the image was taken relative to the meniscus (see the color bar in (**a**)). All images are taken for the same sample orientation as it was pulled out. The white arrow in (**e**) shows the pulling direction.

During the pulling process, the FF crystallization occurred at the air–solution interface, where a meniscus was formed (Figure 1a). In particular, we observed the formation of microcrystals with different geometries at the top part of the meniscus as presented in Figure 1b. The reason for such morphology can be due to FF monomers concentration gradient in the formed thin film, variation in the evaporation rate, and pulling velocity. Below the meniscus, the evaporation rate decreases, and the diffusion of FF monomers becomes capable to provide enough monomers for belt-shape microstructures formation (Figure 1c,d). The growing microbelts are disordered at the beginning, but a few millimeters along from the meniscus, they become ordered and form quasi-regular elongated patterns at the substrate propagating over a millimeters-long distance depending on the size of the substrate (Figure 1e). This area is the most suitable for TENG fabrication.

**Figure 2:**
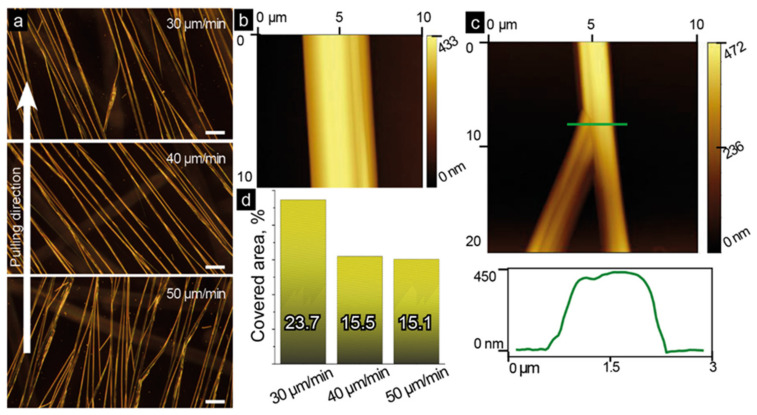
Morphology of quasi-regular belt-like FF microstructures: (**a**) optical dark-field images of belts grown at different pulling velocities (scale bar: 50 µm). All images are taken for the same sample orientation as they were pulled out. The white arrow shows the pulling direction. (**b**) AFM topography image of a microbelt; (**c**) AFM topography image of the microbelt split, and its cross-section along the green line; (**d**) the estimated coverage area for samples pulled with different velocities.

Figure 2a demonstrates optical dark-field images of unidirectional parallel microbelts grown at different velocities of the substrate pulling. The formed regular structures can deviate at about 20° from the pulling direction. AFM analyses revealed that the crystals grown with different pulling velocities have a uniform width of 3.7 ± 1.6 µm and a height varying in the range of 0.5–1 µm; however, the coverage area is different. For the pulling velocity of 30 μm/min, the coverage is about 24%, whereas, for velocities 40 and 50 µm/min, samples show similar coverage of about 15% (Figure 2d). It is worth noting here that lower pulling velocities (e.g., 20 μm/min) lead to growing a net of randomly oriented and overlapping FF microbelts probably due to an excess of the monomers near the slowly pulled substrate that makes the crystallization conditions close to the static regime realized in standard drop casting experiments. Such structures are less suitable for fabricating a TENG, and therefore, they were not considered in our study.

The obtained results are likely related to the crystal splitting taking place during the pulling process. An example of such splitting is demonstrated in Figure 2c. We hypothesize that during the self-assembly process, some defects can be formed initiating splitting and allowing the growth of a new crystal in different directions. Using a cross-section view (Figure 2c), one can observe the second formed crystal with a slightly lower height and a clear edge between the splitting crystals. On the other hand, a straight line can be observed along the non-split crystal (Figure 2b), which supports our assumption that the splitting can be related to the crystal morphology.

### 3.2. Structure Analysis

**Figure 3:**
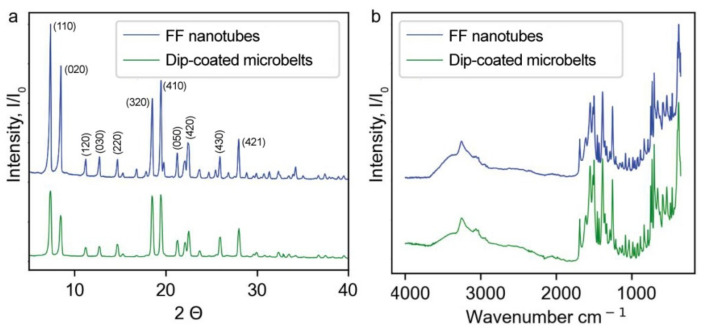
Crystal structure comparison of FF nanotubes and dip-coated microbelts: (**a**) XRD diffractograms, (**b**) FTIR spectra.

The structure of dip-coated microbelts was characterized by XRD and FTIR spectroscopy. Figure 3a shows that the XRD pattern of dip-coated structures well coincides with that of conventional self-assembled FF peptide nanotubes, though several low intense peaks are not clearly seen. FTIR spectra of both samples are identical as well (Figure 3b). These results imply that dip-coated microbelts represent dense bundles of FF nanotubes.

### 3.3. Triboelectric Measurements

**Figure 4:**
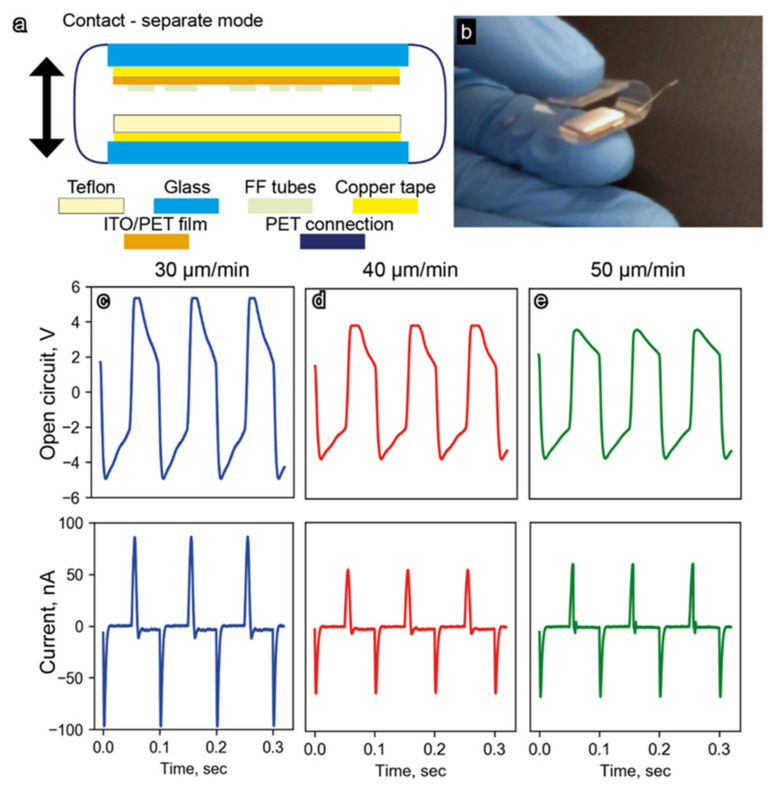
(**a**) Schematic of the developed FF-based TENG; (**b**) full-size image of the TENG prototype used for experimental testing. Output performance characteristics (open-circuit voltage and shortcut current) of the developed TENGs fabricated with FF microbelts grown with different pulling velocities: (**c**) 30 µm/min, (**d**) 40 µm/min, and (**e**) 50 µm/min.

It is known that Teflon is used in TENGs as a negatively charged material [36,37], whereas cyclo-FF structures provide a positive polarity [38]. Therefore, we used 1 × 1 × 0.1 cm^3^ Teflon plates as the second triboelectric body for the FF/ITO/PET substrates. A schematic of the developed contact-separated TENG and its full-size optical image are presented in Figure 4a,b, respectively. Two 15 × 20 mm^2^ glass substrates were used as the outermost layers of the generator. A 20 × 60 mm^2^ PET film was used to combine substrates in a single device. The 10 × 10 × 1 mm^3^ Teflon plate was glued to the inner side of one glass-base by copper tape, whereas the ITO/PET film with a 10 × 12 mm^2^ area covered by dip-coated FF microbelts was adhered to the opposite glass-base by a two-side adhesive tape. The wire electrodes were connected to the copper tape and ITO/PET substrate by the silver epoxy.

When a square-wave voltage was applied to the shaker, similar waveforms with the same frequency and phase were observed for the open-circuit voltage, V_OC_ (Figure 4c–e). At the same time, shortcut current, I_SC_, demonstrated a peak-shaped waveform characteristic of triboelectric generators (Figure 4c–e). The highest values of V_OC_ and I_SC_ around 5 V and 100 nA, respectively, were obtained for the sample with the microbelts grown at a pulling velocity of 30 μm/min. These values are noticeably larger than those for other samples with V_OC_ and I_SC_ around 4 V and 50 nA, respectively. These parameters are significantly higher than those for a piezoelectric nanogenerator based on analogous arrays of FF microtubes (V_OC_ = 2.8 V and I_SC_ = 37.4 nA [26]).

It should be noted that a partial degradation of FF microbelts was observed during the triboelectric measurements; however, TENG’s output performance was quite stable during all measurements. Taking in mind the triboelectric measurements were performed at a frequency of 10 Hz, and the typical measurement duration was 1–2 min, one can conclude that the fabricated FF-based TENG supported at least 1000 cycles. We suppose that further optimization of the TENG geometry and operating conditions would allow for increasing the TENG stability without performance loss.

## 4. Discussion

**Figure 5:**
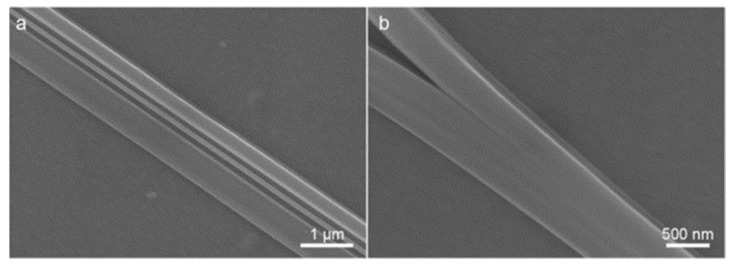
SEM images of FF microbelts: (**a**) three parallel dense packed belts; (**b**) microbelt’s splitting.

The XRD and FTIR measurements showed that the crystal structure of the grown microcrystals is almost identical to that of conventional FF microtubes. These microtubes are apparently composed of helical nanotubes held by means of π-π interaction between aromatic side chains [39]. Microstructures fabricated via dip-coating did not exhibit a traditional hexagonal shape. On the contrary, AFM and SEM images (Figure 2b and Figure 5, respectively) demonstrate flat and wide microbelts or microribbons grown on the substrate. In this work, the FF concentration in water–HFIP mixture is four times lower than the standard one (2 mg/mL) [29]. Kim et al. showed that the variation of this concentration can cause the formation of either microtubes or microrods [40]. However, the spatial limitation related to the delivery of the monomers carried out in the dip-coating meniscus is probably a more significant factor determining the belt-like shape of the microcrystals. During the dip-coating process, the growth of FF microtubes occurs in the thin film of the solution forming a flat meniscus, where the Marangoni flows parallel to the substrate take place [41]. These flows promote the preferable growth of the microstructures at the substrate. The thickness of the solution film limits the height of the grown structures thus leading to the observed flat morphology (Figure 2b and Figure 5). Some microtubes demonstrate a tendency to grow parallel and very tight to each other (Figure 5a), while some of them undergo splitting (Figure 5b).

It was found that not only the morphology of the microstructures is relevant to their usability for triboelectric applications, but also the existence of water molecules in the nanochannels of microtubes [22,42,43]. The effect of air humidity on the triboelectric effect and thus output characteristics of the TENGs was recently demonstrated [44]. Therefore, we examined the effect of the trapped water on the triboelectric charge generated by the developed FF TENG.

**Figure 6:**
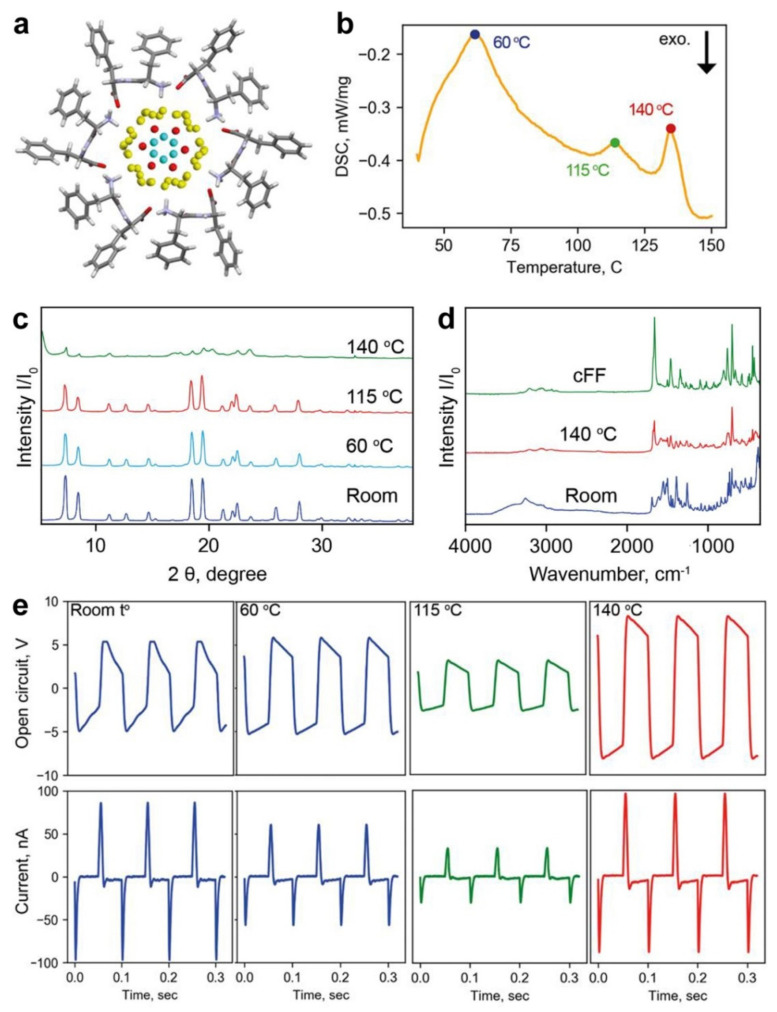
Layered structure of water molecules inside the FF nanochannels: (**a**) reconstruction of the FF nanotubes from X-ray data; yellow spheres denote bound water; red, and cyan spheres—mobile water. (**b**) DSC curve showing three peaks corresponding to evaporation of different types of water. (**c**) XRD patterns of the FF powder heated at different temperatures, and (**d**) FTIR spectra of FF crystals before annealing (room temperature—blue line) and after 140 °C (red line) and cFF (green line). (**e**) Triboelectric output performances Voc and Isc after the TENG treatment at different temperatures.

The reconstruction of XRD data demonstrated two types of water molecules confined in FF nanochannels of 1 nm diameter: sedentary “bound water”, directly interacting with peptide shell; and “mobile water”, localized near the nanotube axis [34,45] (Figure 6a). The existence of these types of water is also confirmed by DSC, where two endothermic peaks (at 60 and 115 °C) corresponding to the evaporation of mobile and bound water, respectively, [34] are observed (Figure 6b). The third peak at 140 °C is obviously due to the peptide cyclization started. Figure 6c shows XRD diffraction patterns of the FF microbelts measured during the heating. For temperatures below 140 °C, the crystal structure remains the same. However, at 140 °C, the intensities of all peaks significantly decrease which is a signature of the sample amorphization. FTIR spectra confirm the crystal structure preservation at temperatures below 140 °C, whereas a cyclization of the microtubes occurs at 140 °C (Figure 6d). The shift of the amide I peak located at 1658 cm^−1^ in the spectrum of the initial sample to 1674 cm^−1^ for the sample heated up to 140 °C demonstrates the unfolding of the α-helical structure into the β-sheet [46]. Thus, summarizing XRD and FTIR results we can conclude that after 140 °C treatment the cyclization of FF molecules and amorphization of the material happen.

To study the effect of water contained in the FF nanotubes on the TENG output performance, a set of FF/ITO/PET samples annealed at the temperatures mentioned above was prepared. Initial samples with the largest belt coverage (around 24%) were used. Figure 6e highlights V_OC_ and I_SC_ measurements. Firstly, note that the temperature dependence of V_OC_ is different than that of I_SC_. The V_OC_ remains constant for samples heated up to 60 °C and it is twice decreased for the sample annealed at 115 °C, whereas I_SC_ demonstrates a gradual decrease with increasing the annealing temperature. This difference in temperature behavior suggests that V_OC_ is influenced by bound water which remains unaffected by heating until 115 °C, whereas the evaporation of mobile water gradually changes the I_SC_. It is reasonable to suggest that molecules of mobile water may provide additional proton conductivity to the microtubes, and their removal obviously reduces the I_SC_. Additionally, the relative permittivity of FF microtubes decreases when water evaporates from the nanochannels [33]. This also can reduce the I_SC_.

**Figure 7:**
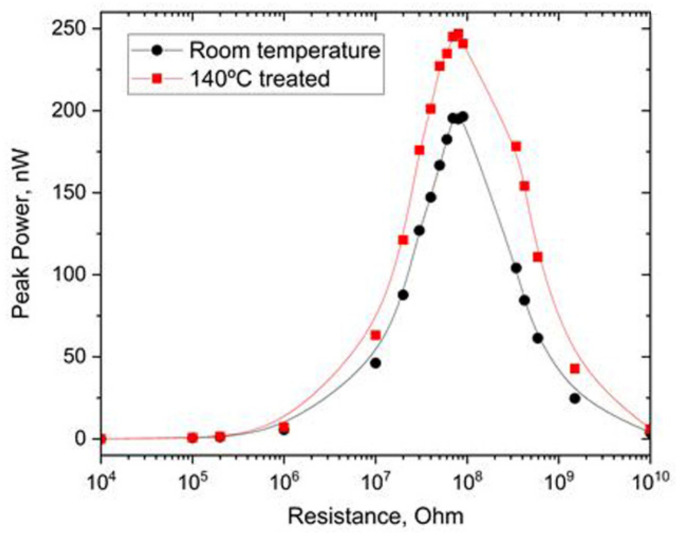
Comparison of the peak power measurements of FF-based TENGs prepared at room temperature and heated to 140 °C.

Both V_OC_ and I_SC_ suddenly increase for the sample dried at 140 °C: the TENG provides I_SC_ similar to those found at room temperature, whereas V_OC_ increased almost twice relative to the initial values (Figure 6e). This was also reflected in the most important characteristic of the TENG—output power (Figure 7). After the annealing at 140 °C, the peak power of the TENG increased from 200 nW (initial structure) to almost 260 nW. This effect is obviously related to the cyclization of the FF molecules and amorphization of the microcrystals [47]. During the cyclization, COO^−^ and NH_3_^+^ groups approach each other and form a new diketopiperazine cycle with water molecule release [48]. Regardless of the disappearance of the charged COO^−^ and NH_3_^+^ groups, the electrostatic interaction continues to play an essential role in cyclo-FF growth on a par with van der Waals interactions [48]. We hypothesize that this increased electrostatic interaction can be responsible for the increased triboelectric response of the cyclo-FF-based TENG. Additionally, the cyclization also leads to the modification of the surface topography. The RMSD roughness of the FF microbelt’s surface (2.267 Å) evaluated using the CSD-Particle tools implemented in the Mercury 2022.2.0 software [49] is about twice as high as that of the surface of the cyclo-FF crystal (1.214 Å). In the latter case, a flatter surface can better interact with the Teflon plate thus providing additional improvement of TENG’s output performance. Further studies will shed more light on the origin of this amplification.

## 5. Conclusions

We used the dip-coating technique to create a quasi-regular structure of aligned FF microstructures at the ITO/PET substrates and used them to construct a contact-separated TENG. Regardless unusual belt-like morphology of the grown structures, their crystal structure corresponds to conventional FF microtubes grown from the solution. We studied the effect of water confined in the nanochannels of FF microtubes on its triboelectric properties. Being a microporous material, FF microtubes contain two types of water molecules inside the nanochannels—bound water strongly interacting with peptide backbone, and mobile water localized near the nanotube axis. We demonstrated that the bound water influences the TENG’s open-circuit voltage, whereas mobile water increases the short-circuit current. We found that the cyclization of FF molecules essentially increases both open-circuit voltage and short-circuit current. This effect is likely attributed to the increased electrostatic interaction in the cyclized sample and the flattening of their surface.

## Data Availability

The data presented in this study are available on request from the corresponding author.
